# Feasibility of Assessing Public Health Impacts of Air Pollution Reduction Programs on a Local Scale: New Haven Case Study

**DOI:** 10.1289/ehp.1002636

**Published:** 2011-02-18

**Authors:** Danelle T. Lobdell, Vlad Isakov, Lisa Baxter, Jawad S. Touma, Mary Beth Smuts, Halûk Özkaynak

**Affiliations:** 1 U.S. Environmental Protection Agency, Office of Research and Development, National Health and Environmental Effects Research Laboratory, Chapel Hill, North Carolina, USA; 2 U.S. Environmental Protection Agency, Office of Research and Development, National Exposure Research Laboratory, Research Triangle Park, North Carolina, USA; 3 U.S. Environmental Protection Agency, Region 1, Boston, Massachusetts, USA

**Keywords:** air pollution, feasibility analysis, health effects, nitrogen oxides, particulate matter

## Abstract

**Background:**

New approaches to link health surveillance data with environmental and population exposure information are needed to examine the health benefits of risk management decisions.

**Objective:**

We examined the feasibility of conducting a local assessment of the public health impacts of cumulative air pollution reduction activities from federal, state, local, and voluntary actions in the City of New Haven, Connecticut (USA).

**Methods:**

Using a hybrid modeling approach that combines regional and local-scale air quality data, we estimated ambient concentrations for multiple air pollutants [e.g., PM_2.5_ (particulate matter ≤ 2.5 μm in aerodynamic diameter), NO_x_ (nitrogen oxides)] for baseline year 2001 and projected emissions for 2010, 2020, and 2030. We assessed the feasibility of detecting health improvements in relation to reductions in air pollution for 26 different pollutant–health outcome linkages using both sample size and exploratory epidemiological simulations to further inform decision-making needs.

**Results:**

Model projections suggested decreases (~ 10–60%) in pollutant concentrations, mainly attributable to decreases in pollutants from local sources between 2001 and 2010. Models indicated considerable spatial variability in the concentrations of most pollutants. Sample size analyses supported the feasibility of identifying linkages between reductions in NO_x_ and improvements in all-cause mortality, prevalence of asthma in children and adults, and cardiovascular and respiratory hospitalizations.

**Conclusion:**

Substantial reductions in air pollution (e.g., ~ 60% for NO_x_) are needed to detect health impacts of environmental actions using traditional epidemiological study designs in small communities like New Haven. In contrast, exploratory epidemiological simulations suggest that it may be possible to demonstrate the health impacts of PM reductions by predicting intraurban pollution gradients within New Haven using coupled models.

Assessing the overall impact of cumulative air pollution programs on environmental public health is a daunting task. Air quality has improved substantially in the United States in recent decades, in large part because of increasingly stringent federal and state air quality regulations. Although many studies have documented links between better air quality and improvements in a variety of human health metrics (Clancy et al. 2002; Friedman et al. 2001; Hedley et al. 2002; Heinrich et al. 2002; Pope 1989; Pope et al. 2009), direct evidence concerning the extent to which specific control measures have improved health is lacking. This lack of evidence is due in part to inherent difficulties in environmental health research concerning effects of relatively low-level exposures on multifactorial health outcomes with long latency periods that often are associated with small changes in relative risk (RR). In addition, complex interactions between interventions over time make it difficult to isolate the environmental health effects of any one regulation.

No single study or study design is likely to characterize the entire range of public health improvements attributable to an air quality regulation (Health Effects Institute 2003). Although the study of the impact of cumulative air pollution control programs on environmental public health is fairly unique compared with those involving individual programs, and formal research approaches are still in early phases of development, several recently published research studies have contributed relevant insights by taking advantage of episodic, one-time events or natural experiments such as a coal ban (Clancy et al. 2002), traffic reductions (Friedman et al. 2001; Tonne et al. 2008), and closure of a steel mill (Parker et al. 2008; Pope 1989). Burnett et al. (2005) proposed a “measure of progress” equivalent to the percent reduction in the RR of adverse health outcomes attributable to reductions in ambient air pollutant concentrations. They note that their proposed approach is applicable to single and multiple cities, as well as single and multiple pollutants. However, they demonstrate their approach only with time-series data on mortality associated with ambient nitrogen dioxide (NO_2_) concentrations from 1981 to 1999 in multiple Canadian cities. Jerrett et al. (2007) pointed out that evidence of short-term health improvements based on studies of “natural experiments” and well-delineated interventions may not apply directly to effects of gradual air quality improvements over many years, and when the follow-up period is long, many other factors, such as random error and systematic biases, especially for low RRs (< 1.05), can obfuscate the linkage of air quality improvements to health benefits. Thus, it is important to determine *a priori* which pollutant–outcome relationships are most likely to result in observable impacts on health in a particular population given projected changes in air pollutant concentrations and estimated risks associated with exposures of concern. Of course, even when benefits are not statistically detectable based on observational data, it is helpful to decision makers to know the circumstances in which health benefits of pollution reductions are expected to occur.

This challenge was recently addressed by Pope et al. (2009) for ambient particulate matter (PM). They used an ecological analytic approach whereby they examined associations between noticeable changes in ambient PM levels and differences in life expectancy estimates [~ 0.61 years per 10-μg/m^3^ decrease in PM ≤ 2.5 μm in aerodynamic diameter (PM_2.5_)] across multiple metropolitan areas in the United States between 1980 and 2000 using multiple regression models adjusted for socioeconomic status, demographic characteristics, and smoking. The models were similar in structure to those previously used in a cross-sectional analysis of air pollution effects on mortality by Özkaynak and Thurston (1987). This approach can be applied at a much smaller geographical or urban scale if the necessary health, air pollution, demographic, and other explanatory data can be accessed or estimated at fine-scale spatial resolution. In the present study, we used both conventional sample-size–based approaches and the strategy presented by Pope et al. (2009) to assess the feasibility of conducting a study on the impact that cumulative air pollution reduction programs may have on environmental public health within a small geographic area, New Haven, Connecticut (USA). We then evaluated the strengths and limitations of these approaches in the context of urban scale assessments. We chose New Haven for this assessment because it was designated as one of two Connecticut counties in nonattainment of the PM_2.5_ standard in 1997 [U.S. Environmental Protection Agency (EPA) 2010], and because a variety of air pollution reduction activities have subsequently been implemented at multiple jurisdiction levels by various federal, state, local, and voluntary actions, including the CARE (Community Action for a Renewed Environment) program (U.S. EPA 2011). National and regional initiatives have resulted in large reductions in ambient nitrogen oxides (NO_x_) from mobile sources in New Haven, and the Northeast region also adopted more stringent vehicle emission standards earlier than did other parts of the United States and had faster fleet turnover. In addition, New Haven has implemented a number of voluntary air pollution reduction activities such as promoting smoke-free homes, use of ultra-low-sulfur diesel fuel, school bus retrofits, solvent reduction workshops, and Tools for Schools (City of New Haven 2004).

## Materials and Methods

The New Haven Study Area is centered in the City of New Haven, Connecticut (population ~ 127,000), and extends to a 20-km radius, encompassing 318 census block groups in New Haven County with an estimated population in 2007 of more than 367,000 people. The City of New Haven is located on the southern coast of Connecticut on New Haven Harbor, which is fed by three rivers (the West, Mill, and Quinnipiac) that discharge into northern Long Island Sound. New Haven lies at the intersection of interstates I-91 and I-95, both major regional expressways that are often congested. In addition, several surface arteries pass through or around New Haven, including Routes 1, 10, 17, 34, and 63. Seaborne traffic passes through the Port of New Haven, a deep-water seaport that attracts a considerable number of barges and associated truck and rail traffic. In addition to several institutional power plants, one power generation facility serves the community. This wide range of emission source categories allows for testing of multipollutant emission control strategies.

We evaluated the overall feasibility of assessing the public health impact of air pollution reduction programs in the City of New Haven by linking projected emissions reductions from overall regulatory actions to estimated detectable health outcome changes. We began by identifying pollutants of interest for New Haven based on the local emissions inventory for the baseline year of 2001 (Weil 2004) and criteria air pollutants. For the present study, we focused on two air pollutants: NO_x_ and PM_2.5_. We also identified health outcomes that have been associated with these pollutants: cardiovascular disease hospitalization and mortality; respiratory disease hospitalization and mortality; chronic obstructive pulmonary disease mortality and hospitalization; and asthma prevalence, diagnosis, and hospitalization.

We then evaluated existing data on ambient level air pollution, emission data, personal exposure data, and health outcome data for the New Haven area. As part of this data inventory evaluation, we assessed the relevance and completeness of data, as well as verification of locations and quantities of emissions from local sources. We then generated emission estimates for NO_x_ and PM_2.5_ based on local emissions sources and the projected impacts of federal, state, and local regulatory reduction activities. We also applied an improved methodology to predict mobile source emissions (Cook et al. 2008).

We first estimated pollutant specific local-scale air concentrations using the U.S. EPA’s AERMOD dispersion model (Cimorelli et al. 2005). This model used information on local emission sources and local meteorological conditions to provide hourly and annual average concentrations at multiple locations corresponding to the weighted centroids of each of the 318 census block groups in the study area. We estimated total NO_x_ and PM_2.5_ concentrations by combining regional background levels, chemically reactive pollutant estimates from the CMAQ (Community Multiscale Air Quality) model, and the AERMOD estimates. We estimated emissions using the baseline year (2001) emissions rates and projected emissions in 2010, 2020, and 2030 based on planned and anticipated pollution control programs.

To assess feasibility using a sample size approach, we first determined the minimum detectable decrease in each outcome relative to its baseline incidence rate [tests of two independent proportions for a (one-sided) likelihood ratio chi-square test with an α of 0.05 and power of 0.80] for a study population of 367,173 (i.e., the 2007 Census estimate for the New Haven population within the 318 block groups included in the study area). For some of the health outcomes, we made additional study area subpopulation calculations for different age groups (< 18 years, ≥ 18 years).

There is general consensus that RRs associated with air pollution exposure for a wide variety of health outcomes are typically less than 1.50, and usually within the range of 1.01–1.20, often for a 10-μg/m^3^ change in PM_2.5_ or an interquartile range change in gaseous pollutant concentrations. Jerrett et al. (2008) and Wellenius et al. (2005) found risk ratios or a RR in this range for NO_2_. RRs for PM_2.5_ and various outcomes in this range were found by Pope et al. (2002) and Sheppard et al. (1999), whereas Laden et al. (2006) found higher PM_2.5_ and mortality RRs of 1.16–1.28, and Peters et al. (2001) found an RR of 1.69 for PM_2.5_ and acute myocardial infarction.

Next we determined the percent reduction in exposure that would be required to produce a given reduction in the outcome assuming a range of possible effect sizes for concentration–outcome associations. Specifically, we considered air pollution RR values of 1.01, 1.05, 1.10, 1.15, and 1.20 representing the increase in outcome (*y*) associated with an incremental increase in a given pollutant exposure equal to the level of the average value of the ambient pollution concentration (*c*) in the study population. The change in the outcome (Δ*y*) associated with a change in exposure (Δ*c*) is a function of the baseline incidence rate (*y*) and the risk coefficient (β) for a one-unit increase in exposure:


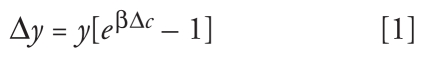


where β = [ln(RR)]/*c*. The percent decrease in exposure (Δ*c*_req_) required to produce a particular reduction in the outcome for a given RR is calculated as


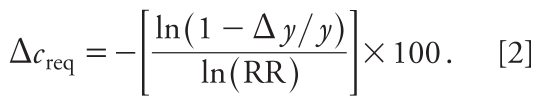


Values of Δ*c*_req_ < 100 indicate the percent reduction in exposure that would be required to produce a specific reduction in the outcome (Δ*y*) assuming a given RR for the exposure–outcome association. Values of Δ*c*_req_ ≥ 100 indicate that the corresponding value for Δ*y* is not feasible, because exposure would have to be reduced by more than 100% to achieve it.

Finally, we combined data on projected changes in mean annual ambient concentrations of air pollutants for 2010, 2020, and 2030 with the information on minimum detectable effect estimates and the percent reduction in exposure required to produce a given effect estimate to identify which air pollutant–health outcome associations (out of 26 possible combinations) would be most feasible for assessment.

For pollutants such as PM where projected reductions were relatively modest (~ 8%), we used an exploratory epidemiological methodology similar to that presented in Pope et al. (2009). Specifically, we used the simulated health data at census block group level (derived from county-specific health data and census information on demographics) to evaluate different strategies for demonstrating impacts of relatively small changes in ambient pollution (compared to NO_x_) over multiple years.

The outcomes for this analysis were the differences between the number of hospitalizations for 2001 and 2010, as illustrated by Equation 3:


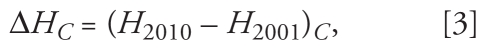


where Δ*H_C_* is the change in the number of hospitalizations at census block group *C* and *H*_2010_ and *H*_2001_ are the number of hospitalizations for the years 2010 and 2001, respectively.

We calculated the number of hospitalizations due to congestive heart disease [CHD; *International Classification of Diseases*, *9th Revision, Clinical Modification* (ICD-9CM; World Health Organization 2004), code 428] and asthma (ICD-9CM, code 493) for each census block group for the years 2010 and 2001. We chose these end points based on significant associations (1.28% increase in risk per 10-μg/m^3^ increase in same-day PM_2.5_) reported by Dominici et al. (2006) between PM and CHD hospitalizations for the Medicare cohort. We restricted hospitalizations due to CHD to the population > 65 years of age, and we calculated asthma hospitalizations separately for all ages and for the population < 25 years of age, because of known age-dependent differences [Connecticut Department of Public Health (CDPH) 2007]. We calculated the number of hospitalizations for the years 2001 and 2010 as

where *H_c_* is the number of hospitalizations for census block group *C*, Rate is the rate of hospitalization for females (*F*) and males (*M*) of race *R* (white, Hispanic, black), and Pop*_C_* is the population of each subgroup (e.g., white female, black male, etc.).


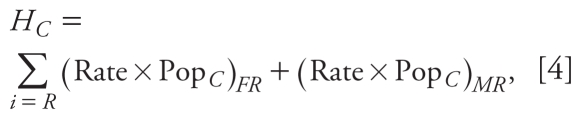


We used 2000 U.S. Census Bureau (2001) data to estimate the size of each population subgroup in 2001. We used county-level population projections for 2010 to estimate the proportional change in each population subgroup from 2000 to 2010 and applied this to the 2000 census block group population to estimate 2010 census block populations for each subgroup.

Hospitalization rates for both outcomes are available for all of New Haven County for 2001 (CDPH 2001) and 2007 (CDPH 2007), and we used 2007 data for 2010 hospitalizations. The rates are broken down by age and sex, and age and race, but not by age, sex, and race. We therefore assumed constant ratios of rates of hospitalizations for males and females for all races. We calculated hospitalization rates according to sex, race, age (> 65 years of age for CHD, all ages, and < 25 years of age for asthma), health outcome (CHD or asthma), and year (2001 or 2010) as


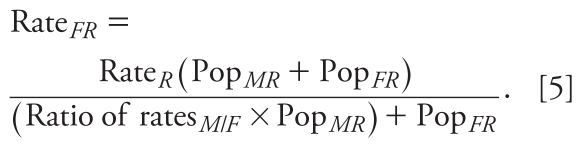


For example, Rate*_FR_* is the county-level hospitalization for females of race *R*; Rate*_R_* is the county-level hospitalization rate for race *R*; Ratio of rates*_M_*_/_*_F_* is the ratio of hospitalization rates between males and females; and Pop*_FR_* and Pop*_MR_* are county-level population sizes for females and males of race *R*, respectively. We then calculated Rate*_MR_* by multiplying Rate*_FR_* by Ratio of rates*_M_*_/_*_F_*.

Reductions of PM_2.5_ at each census block group were then regressed against the changes in hospitalizations from 2001 to 2010 at each census block group. All regression analyses were performed using SAS (version 9.1; SAS Institute Inc., Cary, NC).

Our analysis indirectly accounted for the effects due to changes in key ethnic/racial demographic profile by using group or sex relevant hospitalization rates as part of the health data and feasibility simulations. We explored the influence of introducing an additional explanatory variable in our health effects regressions to indirectly account for both neighborhood effects and the missing determinants of observed hospital admissions by computing average admissions either within a 3- or 4-km radius around each census tract (similarly considered by Özkaynak and Thurston 1987).

## Results

### Air quality modeling

**Figure 1:**
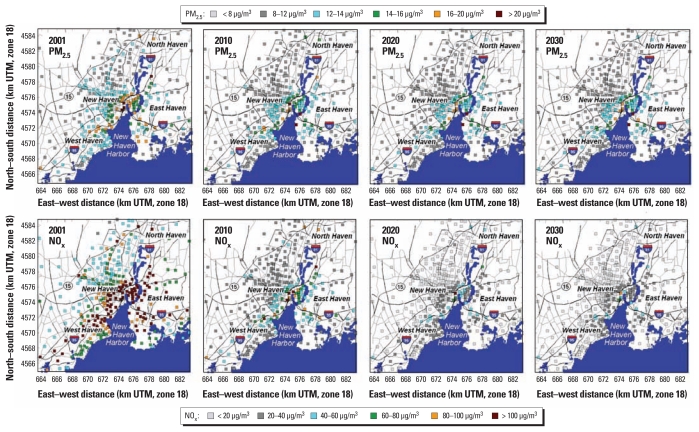
Maps of modeled PM_2.5_ and NO_x_ concentrations for 2001 (baseline), 2010, 2020, and 2030.

We produced combined CMAQ and AERMOD model results for the study area. Model estimates for NO_x_ and PM_2.5_ were consistent with measured Air Quality System (AQS) monitoring data for the area (Johnson et al. 2010). Figure 1 shows maps of modeled PM_2.5_ and NO_x_ concentrations for the baseline year (2001) and projections for 2010, 2020, and 2030. PM_2.5_ maps for all four time points show a wide range of concentrations in the study area, with high concentrations in the city center, near the port areas, and near major roadways such as I-95, whereas PM_2.5_ concentrations in suburban areas were much lower than in central parts of the study area. Finally, PM_2.5_ concentrations are projected to decrease over time, with the most pronounced decreases in areas with the highest estimated concentrations in 2001.

Spatial patterns in ambient air quality concentrations for NO_x_ relates strongly to the sources of mobile emissions such as major highways. NO_x_ concentrations shown here depict strong spatial gradients because many of the locations for which we estimated concentrations (population-weighted centroids of the 318 census block groups) are near major roadways. Thus, contrasts between those areas and suburban areas are very pronounced. Finally, NO_x_ concentrations are also projected to decrease considerably over time, particularly in locations close to roadways, because of the implementation of federal emission standards for mobile source emissions.

**Figure 2:**
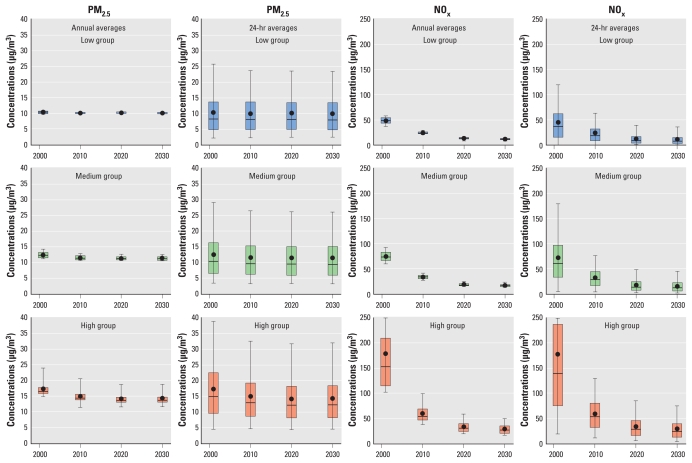
Distributions of annual and daily average modeled PM_2.5_ and NO_x_ concentrations for the baseline model year 2001 and projections for 2010, 2020, and 2030. The distributions for the census block groups are classified into three groups according to annual average PM_2.5_ and NO_x_ concentration distributions in 2001: low, lowest 25%; medium, interquartile range; high, highest 25%.

Figure 2 shows distributions of annual and daily average modeled PM_2.5_ and NO_x_ concentrations for the baseline model year 2001 and projections (2010, 2020, and 2030). We sorted the annual average concentrations for 2001 in order to group the 318 study area locations (census block groups) for which we divided estimates into three groups according to average pollutant concentrations at baseline: low (locations in the lowest 25% of the distribution), medium (locations in the 2nd and 3rd quartiles), and high (locations in the highest quartile of the distribution). As expected, daily averages are more variable than annual averages for both PM_2.5_ and NO_x_, which indicates the importance of temporal variability in pollutant concentrations. Downward trends in PM_2.5_ concentrations were evident for areas with medium and high concentrations, but not for low-concentration areas. Declines in NO_x_ concentrations were evident over time for all three groups, but the decrease was also much sharper in the high-concentration areas. The models predicted large percentage decreases for NO_x_ between 2001 and 2010 (61%) and with less pronounced decreases from 2001 to 2020 and 2030 (overall decreases of 78% and 81%, respectively). For PM_2.5_ the models predicted smaller percentage decreases from 2001 to 2010 (8%), 2020 (9%), and 2030 (9%).

### Sample-size–based feasibility analysis

Table 1 exhibits the percent reduction in concentrations for a given pollutant that would be needed if we assumed a specific RR (1.01, 1.05, 1.10, 1.15, and 1.20) and an estimated reduction in adverse health outcome (2.5%, 5%, 10%, 15%, and 20%) using the assumptions stated above. Table 2 lists the health outcomes that we explored with corresponding minimum statistically detectable decreases in each outcome for the New Haven study population given the baseline rate of the outcome. The New Haven study population area is sufficient in size to examine reductions in adverse health outcomes ranging from a low of 2.5% (adult asthma prevalence) to a high of 10% (all-cause mortality and hospital discharge for cardiovascular diseases and respiratory causes).

Based on the percentage decrease air pollution projected for 2010, 2020, and 2030 within New Haven for NO_x_ (61%, 2010; 78%, 2020; 81%, 2030) and PM_2.5_ (8%, 2010; 9%, 2020 and 2030), the percent reduction in air pollution needed to produce a given change in the outcome (Table 1), and the minimum statistically significant percent decrease in each health outcome that can be detected in the New Haven study population (Table 2), we can assess the feasibility for detecting beneficial health effects of air pollution reductions. Of the 26 different air pollution–health outcome linkages assessed, only five, all NO_x_ related, are potentially feasible (Table 2, last column): all-cause mortality, cardiovascular disease hospitalization, respiratory disease hospitalization discharge, current prevalence of asthma in children, and current prevalence of asthma in adults.

### Simulation-based epidemiological feasibility analysis

**Figure 3:**
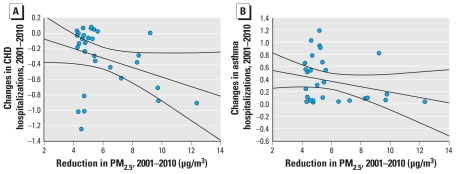
Changes in hospitalizations from 2001 to 2010 according to predicted reductions in PM_2.5_ concentrations based on linear regression (with 95% confidence intervals) among 30 census block groups with a predicted PM_2.5_ reduction > 4 μg/m^3^. (*A*) CHD hospitalizations among those > 65 years of age. (*B*) Asthma hospitalizations (all ages). Dots represent observed data for individual census block groups.

The average number of hospitalizations for CHD among those ≥ 65 years of age in each census block group decreased between 2001 and 2010, whereas average numbers of asthma hospitalizations increased (Table 3). We were unable to detect associations between small reductions in PM_2.5_ pollution concentrations and health outcomes, so we restricted our analysis to census block groups with PM_2.5_ reductions of > 4 μg/m^3^ (*n* = 30). For these census block groups, numbers of CHD hospitalizations were inversely associated with the estimated reduction in PM_2.5_ concentrations, indicating that numbers of hospitalizations decreased as the reduction in PM_2.5_ increased (*p* < 0.1; Table 4, Figure 3A). Asthma hospitalizations were also inversely associated with reductions in PM_2.5_ concentrations based on our simulations, suggesting that greater reductions in PM_2.5_ may slow the increase in asthma hospitalizations over time (Table 4, Figure 3B). However, the inverse association was weaker than for CHD hospitalizations. Finally, an exploratory analysis we conducted by including additional surrogate variables in the regression models aimed at capturing neighborhood effects caused substantial increases in model *R*^2^ values, whereas the PM_2.5_ effect estimates were attenuated somewhat depending on the outcome chosen (data not shown).

## Discussion

We used detailed information on local health and exposure-related data to assess the feasibility of identifying an impact of cumulative air pollution programs on environmental public health in New Haven for 26 different pollutant–health outcome linkages. Combined regional (CMAQ) and local-scale (AERMOD) air quality modeling analysis showed a small overall decrease for PM_2.5_ (~ 8–9%) in mean pollutant concentrations mostly from local sources and between 2001 and 2010; in contrast, we projected that NO_x_ would decrease by > 60%. Most NO_x_ reductions can be attributed to mobile source emission reduction programs. Thus, it is important to accurately characterize near-road impacts. Local reductions in PM_2.5_ are modest relative to high background PM concentrations. Statistical power calculations suggest that projected decreases in NO_x_ may result in statistically significant improvements in health outcomes, including all-cause mortality, asthma prevalence in children and adults, and cardiovascular and respiratory hospitalizations. For other pollutants with more modest reductions, including PM, we determined the likelihood of performing a successful traditional air pollution reduction–health reduction analysis in New Haven to be poor. Alternative epidemiological study designs that use spatially and temporally resolved air quality and exposure models to characterize intraurban gradients were promising based on exploratory epidemiological simulations. However, health outcomes with low baseline rates would have to be strongly associated with air pollution exposures in order for exposure reductions to result in identifiable improvements and thus would not be ideal for examining risk management decisions.

This study illustrates the advantages of using air quality models over traditional epidemiological approaches using ambient measurements. For example, central-site data are especially problematic for certain PM components and species (e.g., elemental carbon, organic carbon, coarse and ultrafine PM) that exhibit significant spatial heterogeneity. Also, for many pollutants (e.g., toxic pollutants), ambient monitoring data are often nonexistent or limited. Appropriately verified air quality models, on the other hand, can provide the needed spatial and temporal resolution for multiple air pollutant concentrations at many locations. These same models can also be used to estimate the projected air quality and inputs for exposure models for future years, dependent on air pollution reduction activities, or due to the addition of new sources in a community (Isakov et al. 2006). For example, this model can address what happens if emissions from some specific stationary or mobile sources are reduced by certain amounts and what the associated impacts of these local controls versus regional controls may be. This model application helps determine which control options are most effective in reducing ambient concentrations.

Both the air quality modeling and feasibility analysis methodologies we used in this research have certain shortcomings. For instance, despite their advantages of being able to provide temporal (hourly) and spatial (at hundreds of locations) estimates, and having a long history of use by regulatory agencies in multipollutant mitigation strategies, models have uncertainties due to model inputs, algorithms, and model parameters (Sax and Isakov 2003). Therefore, in order to reduce uncertainty due to model inputs, detailed emissions and meteorological information should be provided for each model application. In the simulation-based epidemiological feasibility analyses we considered only single-pollutant models and did not include ecological covariates (e.g., income, poverty status, smoking) typically used in cross-sectional, ecological analysis (Özkaynak and Thurston 1987; Pope et al. 2009), because of a lack of complete information. Moreover, it is possible that some of the covariates may change over time, but presumably this may be less of an issue in local-scale assessments than in national-scale analyses. We did not perform joint optimizations with NO_x_ and PM, which could be used to examine more complicated alternative study designs such as census block groups with low reduction levels in NO_x_ but intermediate to high reductions in PM. Clearly, accounting for multipollutant strategies in future assessments will be important in implementing enhanced air pollution–health outcome risk management studies (Mauderly et al. 2010).

The linkages between air quality and exposure models (e.g., with the Stochastic Human Exposure and Dose Simulation Model and Hazardous Air Pollution Exposure Model) in the context of the New Haven study have been examined elsewhere (Isakov et al. 2009). Our biggest challenge has been with accessing geographically and temporally resolved health data in New Haven. Of course, this data gap is often a major challenge in other urban areas as well. Although there was strong local cooperation and local, state, and federal interest in working with the project, better research access to locally relevant health data should be both facilitated and encouraged. Given that the 2010 census has recently been collected and the air quality modeling for 2010 can be performed soon, we hope that the methodology we tested can be implemented in the near future using the actual 2010 local air quality modeling, census, and health data, in order to evaluate the results obtained from this feasibility study by using better databases and more robust models.

Bolstered by the findings from our study, the City of New Haven has been working to find better solutions for reducing air pollution burden and for understanding the impacts from air emissions. We presented the results from this analysis to the New Haven departments of Health, City Planning, and Economic Development and to the city chief executive officer. These results have been used by New Haven in finalizing their negotiations to obtain zero emissions from a proposed new power plant unit to meet peak demand operations, which will be achieved through offsets by the local power plant company and proposed retrofits of garbage trucks and some port operations and additional community benefits. Moreover, the city is also evaluating what can be done to reduce impacts from port operations and mitigate exposures at city schools located near busy roads and highways, in light of the detailed air quality modeling results and health risk evaluations presented here.

## Conclusions

In this project we successfully applied, compared, and evaluated exposure assessment and epidemiological modeling tools in the context of observed public health status in a relatively small community, New Haven, Connecticut, and provided the U.S. EPA and local, state, and city organizations with a new modeling-based methodology to measure the impact of collective risk mitigation approaches and regulations. Furthermore, because no single regulation or program that affects air quality can be isolated to track its effect on health, this project provided critical findings on how regulatory agencies may better examine the complex interactions of cumulative impacts on air quality and health effects from multiple actions in other urban communities.

## Figures and Tables

**Table 1:** Percent reduction in air pollution needed to reduce the risk of an outcome by 2.5–20% for a range of assumed RRs.^a^

Percent reduction in outcome	Assumed RR				
	1.01	1.05	1.10	1.15	1.20
2.5	> 100	52	27	18	14
5	> 100	> 100	54	37	28
10	> 100	> 100	> 100	75	58
15	> 100	> 100	> 100	> 100	89
20	> 100	> 100	> 100	> 100	> 100

a Estimates based on Equation 2; the percent reduction in outcome corresponds to Δ*y*/*y*, and the estimated values for percent reduction in air pollution required correspond to Δ*c*_req_. The Δc_req_ value ≥ 100% indicates that the corresponding value for Δ*y*/*y* is not feasible, because exposure would have to be reduced by more than 100% to achieve it.

**Table 2:** Minimum statistically detectable difference in health reduction for a given reference rate and affected population, based on sample size calculations.

Outcome	Reference rate (per 100,000)	Reference rate source information	New Haven Study Area estimated 2007 population	Minimum statistically detectable percent decrease	NO_x_ feasibility based on sample size^a^
All-cause mortality (except injury)	812.9	State of Connecticut, 1999–2006 (Connecticut age-adjusted rate)^b^	367,173	10	2020, 2030 (RR ≥ 1.15); 2010 (RR = 1.20)

Cardiovascular disease					

Mortality (ICD-10 codes I00–I87)	316.9	State of Connecticut, 1999–2006 (Connecticut age-adjusted rate)^b^	367,173	15	Not feasible
Hospitalization discharge (ICD-9 codes 390–459)	1059.67	County of New Haven, 2006 (Connecticut age-adjusted rate)^c^	367,173	10	2020, 2030 (RR ≥ 1.15); 2010 (RR = 1.20)

Respiratory disease					

Mortality (ICD-10 codes J00–J98)	89.5	State of Connecticut, 1999–2006 (Connecticut age-adjusted rate)^b^	367,173	20	Not feasible
Hospitalization discharge (ICD-9 codes 460–519)	948.05	County of New Haven, 2006 (Connecticut age-adjusted rate)^c^	367,173	10	2020, 2030 (RR ≥ 1.15); 2010 (RR = 1.20)

Chronic obstructive pulmonary disease and related disorders					

Mortality (ICD-10 codes J40–J44)	40.7	State of Connecticut, 1999–2006 (Connecticut age-adjusted rate)^b^	367,173	30	Not feasible
Hospitalization discharge (ICD-9 codes 490–496)	266.81	County of New Haven, 2006 (Connecticut age-adjusted rate)^c^	367,173	15	Not feasible

Asthma					

Current prevalence, adults (≥ 18 years of age)	7,900	County of New Haven, 2006^d^	283,232	2.5	2010, 2020, 2030 (RR ≥ 1.05)
Current prevalence, children (< 18 years of age)	8,800	County of New Haven, 2006^d^	83,941	5	2010, 2020, 2030 (RR ≥ 1.10)
Hospitalizations (ICD-9 code 493), adults (≥ 18 years of age)	314	City of New Haven, 2001–2005^e^	283,232	15	Not feasible
	144	County of New Haven, 2001–2005^e^	283,232	20	Not feasible
Hospitalizations (ICD-9 code 493), children (< 18 years of age)	716	City of New Haven, 2001–2005^e^	83,941	15	Not feasible
	290	County of New Haven, 2001–2005^e^	83,941	25	Not feasible

ICD-9 (World Health Organization 2004); ICD-10 (World Health Organization 2007).

a Based on Table 1, the minimum statistically detectable percent decrease in this table, and the forecasted reductions in NO_x_ 2010 (61%), 2020 (78%), and 2030 (81%). An air pollutant–health outcome combination is considered feasible for assessment of air pollution reductions if the reduction in an air pollutant for a given health minimum statistically detectable percent decrease is within the range of given RRs.

b Data from the Centers for Disease Control and Prevention (2009).

c Data from the CDPH (2006).

d Connecticut Behavioral Risk Factor Surveillance System, 2006 (see Peng et al. 2008).

e Office of Health Care Access Discharge Database (see Peng et al. 2008).

**Table 3:** Distribution of estimated changes in CHD and asthma hospitalizations among 318 New Haven census block groups, 2001–2010.^a^

Outcome^b^	Mean ± SD^c^	Interquartile range	Range
CHD hospitalizations for the population ≥ 65 years of age	−0.52 ± 0.56	−0.77 to −0.13	−4.11 to 1.07
Asthma hospitalizations for all ages	0.23 ± 0.27	0.04 to 0.31	0.003 to 1.25
Asthma hospitalizations for the population < 24 years of age	0.13 ± 0.18	−0.003 to 0.22	−0.03 to 1.20

a Hospitalizations in 2010 are represented by 2007 hospitalization data.

b Number of hospitalizations in 2010 minus number of hospitalizations in 2001 for each census block group.

c Negative values indicate a decrease in the average number of hospitalizations across census block groups from 2001 to 2010; positive values, an increase.

**Table 4:** Associations of predicted reductions in PM_2.5_ with changes in CHD hospitalizations and in asthma hospitalization among 30 New Haven census block groups with > 4-μg/m^3^ decrease in average PM_2.5_ concentrations, 2001–2010.^a^

Outcome	*R*^2^	β (95% CI)^b^
Change in CHD hospitalizations among those ≥ 65 years of age	0.08	−0.06 (−0.13 to 0.01)*
Change in asthma hospitalization (all ages)	0.03	−0.04 (−0.11 to 0.02)
Change in asthma hospitalization for children < 24 years of age	0.01	−0.02 (−0.05 to 0.01)

CI, confidence interval.

a Hospitalizations in 2010 are represented by 2007 hospitalization data for 30 census block groups with a predicted decline in PM_2.5_ of 4 μg/m^3^ or more between 2001 and 2010.

b Linear regression coefficient (95% confidence interval) for the association between hospitalizations and the decline in PM_2.5_ between 2001 and 2010.

* *p* < 0.1.
